# Participant perspectives on the acceptability and effectiveness of mindfulness-based cognitive behaviour therapy approaches for obsessive compulsive disorder

**DOI:** 10.1371/journal.pone.0238845

**Published:** 2020-10-21

**Authors:** Tamara Leeuwerik, Kate Cavanagh, Elizabeth Forrester, Claire Hoadley, Anna-Marie Jones, Laura Lea, Claire Rosten, Clara Strauss

**Affiliations:** 1 School of Psychology, University of Sussex, Brighton, United Kingdom; 2 Independent Consultant, London, United Kingdom; 3 Sussex Partnership NHS Foundation Trust, Brighton, United Kingdom; 4 School of Health Science, University of Brighton, Brighton, United Kingdom; Boston University, UNITED STATES

## Abstract

Cognitive behavioural therapy (CBT) which includes Exposure and Response (ERP) is a highly effective, gold standard treatment for Obsessive-Compulsive Disorder (OCD). Nonetheless, not all patients with OCD significantly benefit from CBT. This has generated interest in the potential benefits of Mindfulness-Based Interventions (MBIs), either integrated with CBT, to enhance engagement with ERP tasks, or delivered as a stand-alone, first-line or therapy to augment CBT. This paper reports on two qualitative studies that involved a thematic analysis of interview data with participants in a 10-week Mindfulness-Based ERP (MB-ERP) course (study 1) and a 9-week Mindfulness-Based Cognitive Therapy course adapted for OCD (MBCT-OCD) (study 2). Whilst MB-ERP integrated a mindfulness component into a standard ERP protocol, MBCT-OCD adapted the psychoeducational components of the standard MBCT for depression protocol to suit OCD, but without explicit ERP tasks. Three common main themes emerged across MB-ERP and MBCT-OCD: ‘satisfaction with course features’, ‘acceptability of key therapeutic tasks ‘and ‘using mindfulness to respond differently to OCD’. Sub-themes identified under the first two main themes were mostly unique to MB-ERP or MBCT-OCD, with the exception of ‘(struggles with) developing a mindfulness practice routine’ whilst most of the sub-themes under the last main theme were shared across MB-ERP and MBCT-OCD participants. Findings suggested that participants generally perceived both MBIs as acceptable and potentially beneficial treatments for OCD, in line with theorised mechanisms of change.

## Introduction

Obsessive-compulsive disorder (OCD) is a debilitating mental health condition characterised by persistent intrusive thoughts, images or urges that cause significant anxiety or discomfort, and repetitive, ritualistic behaviours (e.g. hand washing) or mental acts (e.g. repeating special words) aimed at reducing anxiety or preventing anticipated adverse consequences of the intrusions [1].

The treatment of choice for OCD is exposure and response prevention (ERP), delivered with or without added cognitive strategies [2, 3]. ERP is a form of behaviour therapy that involves patients exposing themselves to their OCD triggers whilst refraining from compulsive behaviours, resulting in habituation [4] and/or new learning that inhibits the existing conditioned response [5, 6]. Cognitive strategies aim to re-evaluate maladaptive appraisals of intrusions (e.g. [7]), as they derive from the cognitive model of OCD that posits that a person with OCD attributes significant meaning to common intrusive thoughts (e.g. [8]) due to maladaptive beliefs such as inflated responsibility, intolerance of uncertainty, perfectionism, overestimation of threat, the need to control thoughts and the over-importance of thoughts [9].

ERP is not a panacea; approximately 30–35% of people do not experience a statistically reliable reduction in symptoms post-treatment whilst 50–55% do not experience remission [10]. Clinically, poor insight, i.e. highly overvalued ideation, OCD symptom severity and comorbid depression and/or anxiety are thought to be associated with poor outcomes (e.g. [1, 11, 12]). However, research evidence to date is equivocal as to predictors of treatment outcomes [12, 13].

ERP is often seen as a challenging therapy by clients and therapists alike [12, 14]; the therapy is anxiety-provoking by design and this is magnified by high levels of distress intolerance associated with OCD [15]. An observational study of OCD over a two-year period found that around 20% of participants who had refused (26%) or dropped out (31%) from CBT for OCD primarily did so due to fears about the treatment [16]. This may also contribute to non-response among treatment completers as patient engagement with between-session ERP tasks is variable [17] and research shows that only a high level of patient engagement is associated with remission [18].

The variable response rate for ERP for OCD informed the exploration of cognitive therapy (CT) (i.e. without ERP) [19, 20]. Most approaches to CT include behavioural experiments to gather evidence to evaluate the accuracy of existing and alternative interpretations of intrusions [21]. This may involve exposure to a trigger and prevention of compulsive behaviour but does not involve systematic and prolonged ERP.

More recently, Jacoby and Abramowitz [22], proposing an inhibitory learning approach to ERP for OCD to maximise treatment outcomes, stressed the importance of patients being supported to develop ‘open-mindedness’ (p.32) towards the experience of anxiety and fear during ERP. This notion is also reflected in an emerging interest in the potential of innovative (add-on) interventions such as meta-cognitive therapy (MCT) [23–25], Acceptance and Commitment Therapy (ACT) [26], and mindfulness-based cognitive therapy (MBCT) for OCD [27, 28]. We suggest that a mindfulness-based approach could facilitate engagement with ERP tasks by enabling greater acceptance of intrusive thoughts, greater tolerance of distress, increased self-efficacy in relation to ERP task initiation and completion and greater self-compassion in response to intrusion-related shame.

Mindfulness can be defined as ‘paying attention in a particular way: on purpose, in the present moment, and nonjudgmentally’ ([29], p. 4). Meta-cognitive therapy and ACT can be conceived of as mindfulness-*informed* interventions; they share theoretical underpinnings with mindfulness-*based* interventions (MBIs), such as Mindfulness-based stress reduction (MBSR) [30] and MBCT [31] and include some mindfulness meditation practice or exercises in their approach. However, they do not include the ‘systematic and sustained training in formal and informal mindfulness meditation practices (for both teacher and participants)’ that characterises MBIs [32]: p. 991) (also see [33]).

Likewise, cognitive therapy and MBCT share much common ground, reflecting both theoretical and procedural overlap [34, 35], but clear points of divergence include that MBCT primarily teaches these skills through mindfulness practice whilst CT uses cognitive restructuring and behavioural experiments. Whilst cognitive therapies can include cognitive strategies that instruct patients to treat intrusions like the background noise of a turned-down radio [21] and teach them to ‘do nothing’ in response to intrusions, i.e. drop compulsions [36], these are mindfulness-*informed* strategies rather than the sustained mindfulness meditation practice that characterises MBCT; daily lengthy mindfulness practice per se is seen as key to successful MBCT outcomes [37]. Also, whilst CT works towards specific goals, MBIs such as MCBT embrace non-striving as participants are invited to ‘simply to observe whatever is happening in each moment without judging it’ [34], p.130) and do not attempt to explicitly target and change the content and meanings attributed to thoughts and/or associated beliefs; rather, they invite participants to observe their thoughts without judgment, teaching them to relate differently to the process of thinking [34, 35].

Research evidence to date suggests that MBIs are effective at reducing symptoms of depression and, to a lesser extent, anxiety [38, 39] and reduce the risk of relapse for depression [40]. There is some research evidence that MBIs achieve their positive effects by enhancing mindfulness and self-compassion and reducing worry, rumination, the suppression or avoidance of negative thoughts, feelings or physical sensations and through altering emotional and cognitive reactivity, e.g. [41, 42].

It is theoretically plausible that MBIs may also benefit OCD, through helping patients to: i) allow intrusive thoughts, images and urges into awareness and bring an interested, accepting attitude to this mental content and associated distress; ii) invite a non-judgmental, de-centred perspective on thoughts as passing mental events rather than facts [31], and; iii) perceive a wider range of choices about how to respond to intrusive thoughts and feelings of anxiety, rather than to react habitually (e.g. compulsions) [43]. In these ways, mindfulness may also facilitate engagement with ERP by enabling greater acceptance and tolerance of unpleasant thoughts, feelings and physical sensations that arise during ERP tasks (e.g. [39, 44–46]). This is an important area to explore as only a high degree and quality of engagement with ERP appears to predict post-treatment symptom remission, e.g. [18]. MBIs also cultivate self-compassion, which may further help to reduce conviction in obsessive beliefs (e.g. about the importance of thoughts or perfectionism) and allow greater acceptance towards feelings of guilt and shame associated with OCD [47, 48].

Quantitative studies of MBIs for OCD include a pilot RCT comparing standard ERP with Mindfulness-based ERP (MB-ERP), which integrates mindfulness practice with a standard ERP protocol [49]. The 95% confidence interval for the post-treatment between-group difference in OCD symptom reduction did not include the 5-point minimum clinically important difference in favour of MB-ERP, suggesting that a larger trial would be unlikely to show that MB-ERP outperformed ERP in terms of OCD symptom reduction. Although results suggested that the addition of the mindfulness component would not likely improve OCD symptom outcomes, the authors concluded that further research would need to ascertain the effects of MBI formats that cultivate mindfulness more intensively. MBCT is a well-established MBI that was designed as a group therapy for depressive relapse prevention, integrates teaching mindfulness skills with psychoeducation, strategies and exercises drawn from cognitive behavioural therapy (CBT) for depression [31].Two recent RCTs of MBCT adapted for OCD as an augmentation therapy for CBT showed small benefits relative to waitlist controls [27] and psychoeducation [28]. A further uncontrolled study of MBCT as a first-line therapy for OCD suggested potentially moderate benefits of MBCT, e.g. [50], although this warrants further investigation using an RCT design that compares MBCT against CBT.

Understanding mechanisms of change in therapies helps to identify, modify and optimise key treatment components and aids the identification of suitable patients for whom the treatment is likely to be beneficial [51, 52]. This is particularly true for multicomponent psychological therapies where effective outcomes could be due to one or more components whilst other components may not enhance outcomes and could be omitted. So far, however, quantitative studies of MBIs for OCD have not formally tested the theorised mechanisms of change and do not necessarily elucidate the perceived acceptability of different adaptations of MBIs. Qualitative research can help to elucidate potential change mechanisms through detailed exploration of participants’ experience of the intervention and their accounts of their own change processes [53–55]; findings could aid change mechanism theory development and refinement [56]. Guidance by the Medical Research Council on process evaluation of complex interventions also highlights that qualitative research, including through participant interviews, can play a crucial role in developing an understanding of the mechanisms of change of new interventions [54]. Therefore, qualitative studies could contribute preliminary evidence towards the acceptability and possible mechanisms of change of adapted MBIs for OCD but they are few and far between. Hertenstein et al. [57] and Sguazzin, Key, Rowa, Bieling, and McCabe [58] conducted qualitative analyses of interviews with patients who had previously completed CBT (including ERP) but continued to experience (residual) OCD symptoms. While participants generally perceived the treatment as acceptable and beneficial, their accounts do not necessarily elucidate whether MBIs might provide a viable treatment for patients who have dropped out from or did not wish to opt-in to CBT. Fairfax, Easey, Fletcher, and Barfield [59] conducted a thematic analysis of brief interviews to explore patient perspectives on a routinely delivered treatment combining mindfulness with CBT. Their findings also suggest a mindfulness approach could potentially enhance CBT in acceptable ways. However, their report did not provide a more in-depth exploration of patients’ experiences and involved secondary care patients, which may not necessarily reflect the experiences of the majority of patients who access treatment for OCD through primary care services.

### Research aims & objectives

This paper reports on two qualitative studies that explored patient perspectives on the acceptability and potential benefits of MBIs for OCD, specifically MB-ERP [49] (study 1) and MBCT adapted for OCD (MBCT-OCD) (study 2). MB-ERP was based on ERP as the primary vehicle of change but included a mindfulness skills training component aimed at enhancing engagement in ERP to improve outcomes. Study 2 followed on from study 1, after the pilot RCT data suggested that MB-ERP would be unlikely to improve on ERP in reducing OCD symptoms post-treatment [49]. Study 2 involved an adapted course of MBCT for OCD, positing mindfulness skills training as the primary vehicle of change in OCD symptoms; it did not involve ERP and included longer and more intensive mindfulness practice than MB-ERP. Unlike previous qualitative studies of MBCT as an augmentation therapy for CBT for OCD [57, 58], study 2 explored whether MBCT-OCD was perceived as an acceptable and beneficial treatment for patients who did not wish to engage with (further) CBT and/or had achieved insufficient benefit from CBT. This was informed by the fact that there are no alternative recommended psychological therapies on offer for such patients [2, 3]. Therefore, it is important to explore viable alternatives, including MBIs for OCD.

This paper reports the findings from each study in turn and then brings these findings together to facilitate an understanding of common and unique experiences associated with these different approaches to teaching mindfulness skills to patients with OCD. The qualitative exploration of patient perspectives on the acceptability and potential benefits of the MB-ERP intervention (study 1) would also help to contextualise the finding that MB-ERP did not appear to improve on ERP outcomes [49].

## Materials and methods study 1

### Design and procedure

This study reports on the thematic and content analysis of semi-structured interviews with MB-ERP participants at six-month post-intervention, conducted as part of a pilot RCT comparing group ERP to group MB-ERP for OCD (see [39, 49] for further details). The pilot RCT was pre-registered (ISRCTN52684820. Registered on 30 January 2014).

All interviews were conducted on NHS premises by a research assistant blind to the group allocation. Interviews were audio-recorded and lasted 30–60 minutes. The interviews were transcribed verbatim and anonymised by CH, a clinical psychology masters’ student and TL, an experienced clinical psychologist and doctoral researcher. This research project was given full ethical approval by an NHS Research Ethics Committee.

### Participants

Participants in the pilot RCT were recruited through two Improving Access to Psychology Therapy (IAPT) services (a primary care public health talking therapies service) in a National Health Service (NHS) mental health Trust in the South of England. Inclusion criteria were: i) 18 + years of age; ii) met DSM-IV diagnostic criteria for OCD [60] based on the Mini International Neuropsychiatric Interview [MINI 6.0.0] [61]; iii) if on psychiatric medication, stable dosage for a minimum of 3 months prior to commencement of the therapy; iv) no plans to change psychiatric medication during the study course; v) had not received any psychological therapy in the 3 months before the current study, nor planned to engage in psychological therapy during the study course. Exclusion criteria were: i) identified organic cause for OCD symptoms; ii) a diagnosed learning disability, psychotic disorder, post-traumatic stress disorder, anorexia nervosa, alcohol dependence or substance addiction; iii) hoarding-only compulsions (see [39, 49] for further details).

Fourteen (74%) of the 19 participants randomly allocated to MB-ERP completed the semi-structured interview. All participants taking part in the pilot RCT were invited and supported to take part in an interview, even if they dropped out of the group. Five participants declined the offer of an interview or attempts to contact them were unsuccessful. Four out of the five participants had dropped out of MB-ERP, for reasons including family or marital problems, pregnancy, difficulty getting time off work and childcare commitments.

All participants had a diagnosis of OCD at the start of treatment (see above). Mean depression severity of the sample was in the moderate range (*M* = 26.93, *SD* = 11.18), as measured with the Beck Depression Inventory-Second edition (BDI-II) [62]. See Table 1 for further sample characteristics.

**Table 1:** Sample characteristics for study 1 (MB-ERP) (N = 14).

Variable		*M(SD)*	*n* (%)
Age (years)*		34.57 (8.28)	
Age of onset (OCD)*		16.07 (6.87)	
Female			11 (79)
White British			13 (93)
Education	Up to secondary (≤ 12 years education)		10 (71)
	Higher education (university, 12+ years)		4 (29)
Employment	(Self-)employed		7 (50)
	Unemployed		7 (50)
On psychotropic mediation at baseline			7 (50)
Prior CBT (self-reported)	Started		6 (43)
	Completed (at least 1 course of NHS routine treatment for OCD)		3 (21)
No of MB-ERP sessions attended		8.00 (3.00)	
		range: 1–10	
Dropout from MB-ERP			2 (14)^a^

Note:

^a^ = after 1–2 sessions.

### Interview schedule

The semi-structured Change Interview [63] was designed to ask participants about their experience of a psychological intervention. The first two sections of the Change Interview ask participants to describe up to five changes they noticed over the course of therapy and to rate, on a 5-point scale, the extent to which they were surprising (1 = ‘very much expected the change to happen’ to 5 = ‘very much surprised by the change’), likely to have occurred without the therapy (1 = ‘very unlikely without the therapy course’ to 5 = ‘very likely without the therapy course’) and important (from 1 = ‘not at all important’ to 5 = ‘extremely important’). Subsequent questions invite participant attributions for these changes (Q3), perceived beneficial or unhelpful aspects of the treatment (Q4 and Q5), and personal and social resources (Q6) and limitations (Q7) to aid therapy engagement, before concluding by inviting participants to suggest ways to improve the course and/or the research (Q8) (see S1 Appendix for interview schedule). The Change Interview was therefore well-suited to the qualitative exploration of participant perspectives on the acceptability and potential benefits of MB-ERP.

### Intervention: MB-ERP

The first of the 10 weekly, two-hour sessions of MB-ERP introduced the rationale for ERP and the inclusion of mindfulness principles and practice. Subsequent sessions (2–10) began with a 10-minute mindfulness practice followed by a 20-minute inquiry into “participants’ direct experience of meditation practices, and exploration of pleasant and unpleasant experiences, which has implications for recognizing established patterns of reactivity and the possibility of responding differently”([32]:p.994-995), with a view for new learning to support participant engagement in ERP. Mindfulness practices included mindfulness of; i) the breath and body (session 1), ii) breath, body, sounds and thoughts (sessions 2–3); iii) intrusive thoughts (session 4–5), and: iv) the body, (intrusive) thoughts, urges and action (sessions 6–10). Verbal guidance for the mindfulness practice was developed by an expert in MBIs and OCD. A three-minute daily mindfulness breathing space practice [31] was also taught from session 6. Practice guidance explicitly invited participants to notice intrusive thoughts, bodily sensations associated with intrusive thoughts/anxiety and compulsive urges and to bring a sense of acceptance to these experiences. Following the mindfulness practice and inquiry, the remaining 90 minutes of each session followed an adapted standard in-vivo ERP protocol (Van Noppen, Steketee, & Pato: Group Behaviour Therapy Treatment Manual for Obsessive Compulsive Disorder, unpublished), including psychoeducation about OCD, planning and reviewing between-session ERP practice and designing and completing within-session ERP. The approach to ERP was adapted to incorporate more recent recommendations derived from inhibitory learning theory [5, 64]. Furthermore, therapists invited participants to apply learnt mindfulness skills to ERP tasks. Session 10 focused on consolidating learning from the therapy. Participants were asked to complete between-session ERP tasks and formal mindfulness practices daily, using provided audio-recordings, and to cultivate mindfulness during daily routine activities. Sessional engagement data, collected for the pilot RCT, showed that participants practised ERP tasks a mean of 16 times a week (*SD* = 8.78, range = 3.78–34.00) (based on 58% completed home practice logs) and completed 4 weekly formal mindfulness practices during the course (*SD* = 0.60, range = 3.67–5.00) (based on 57% completed logs). At the time of the interview, 32% of the participants continued to engage in (some) mindfulness practice.

Two clinical psychologists facilitated the course, one of whom (CS) was an accredited CBT therapist and accredited MBCT teacher. Supervision was provided for both group facilitators by an expert in ERP. Mindfulness supervision was provided by an accredited MBCT supervisor. A lived experience advisory panel provided consultation on the development and implementation of MB-ERP.

### Data analysis

The first two sections of the Change interview, which invite participants to list up to five changes they noticed over the course of therapy and to rate each change for expectedness, importance and likelihood the change would have occurred without the course on 5-point Likert scales, was analysed to explore the perceived (value of) benefits stemming from MB-ERP. Content analysis, which reports frequencies of important content categories in qualitative data, was well-suited to analysing this interview data [65]. All changes reported by participants were collated into an Excel sheet. Low-level categorisation of these changes was conducted, achieved through group consensus (TL, KC, CS), using conventional content analysis [66]. Descriptive statistics were used to report the frequency (*n*, *%*) of each change, along with the average rating (*M*, *SD*) of expectedness, likelihood and importance for each change category.

The remainder of the interview (sections 3–8, see S1 Appendix) invited participants to reflect on attributions for perceived changes, helpful and unhelpful aspects of therapy and personal and social resources and limitations that affected personal engagement with therapy. This data was analysed using reflexive thematic analysis (TA) [67, 68] as it allowed a rich exploration of the acceptability of the therapy course, benefits and potential mechanisms of change from the participants’ point of view, capturing commonalities and differences between participant accounts. If participants discussed these issues in response to the first two sections of the interview, their reflections were included in the TA. Only interview extracts relating to the mindfulness component were included in the analysis, e.g. participants’ reflections on psychoeducation and struggles, engagement in and satisfaction with ERP that did not incorporate a reflection on mindfulness were excluded.

Reflexive TA consists of six phases [68], set out in Table 2. TL conducted phases i. to iii. and vi. of the analysis. The research team (TL, CS, KV) completed phases iv-v together. Making decisions as a group, whereby the different perspectives of multiple researchers converged in a process of mutual confirmation, served as a credibility check [69]. NVivo 12 software was used to conduct the analysis.

**Table 2:** Analytic phases of reflexive thematic analysis.

Phase	Description
i. Familiarising yourself with your data	In-depth familiarisation with the data through repeated reading of all interview transcripts
ii. Generating initial codes	Application of initial codes (i.e. single units of meaning) to transcript extracts that are pertinent to the research question
iii. Searching for themes	Initial codes are examined for commonalities and differences and clustered into overarching themes, and potential sub-themes within these.
iv. Reviewing themes	Themes and sub-themes are reviewed in relation to the coded extracts that supported them to ensure that each theme/sub-theme related to the coded extracts and the whole data set, confirming that the aim of the investigation is maintained and that the research question is answered appropriately
v. Defining and naming themes	The themes’ and sub-themes’ names, descriptions and relationships within the data are finalised
vi. Producing the report	Analysis continues into the write-up of the study report, whereby themes are related back to the research question. The most representative extracts are selected for inclusion in the write-up.

## Results study 1

### Thematic analysis

Table 3 provides an overview of the (definitions of the) main and sub-themes that were developed through the reflexive thematic analysis. Associated sub-themes are discussed below. Pseudonyms were used to protect participant confidentiality.

**Table 3:** Main themes and sub-themes for MB-ERP and MBCT-OCD.

Common (sub-)themes			Unique sub-themes	
Main themes	Captures…	Sub-themes	MB-ERP	MBCT-OCD
Satisfaction with course features	positive and negative perceptions of the course features		Combining mindfulness and ERP	Mindfulness approach
				MBCT compared to CBT
				Course and session structure
				Workbook & forms
				Therapist embodiment of mindfulness
Acceptability of key therapeutic tasks	how participants engaged with the core aspects of the course, specifically mindfulness practice and/or MB-ERP	(Struggles with) developing a mindfulness practice routine		Preference for type of formal practice
				Comparing practice at home and in-session
				(Noticing) reactions to practice
Using mindfulness to respond differently to OCD	how participants perceived mindfulness helped them to respond differently to OCD	Calm and relaxation		Noticing and awareness
		Coming back to the present		Kindness to self
		Observing and allowing		
		Giving less meaning to intrusions		

#### Theme 1: Satisfaction with course features

*Combining mindfulness and ERP*. Several MB-ERP participants reported finding mindfulness meditation beneficial, e.g. describing it as ‘*nice*’ *(Louise)*, ‘*really useful’ (John)*, and ‘*really good’ (George)*. A few were pleasantly surprised by the treatment rationale: ‘*I had never thought of the idea of […] using mindfulness to […] deal with the anxiety that results or to […] help you to do it in the first place*.’ *(George)*. The combination of mindfulness with ERP was perceived as credible and superior to either treatment alone.

#### Theme 2: Acceptability of key therapeutic tasks

*(Struggles with) developing a mindfulness practice routine*. Whilst several participants really valued the (10-15-minute) mindfulness practices, practical barriers to (regular) home practice included finding (uninterrupted) time and space, often due to juggling other commitments, including childcare. The latter meant having to practise in the evening when ‘*it would just make me feel really sleepy’ (Sarah)*. A few participants did not want daily formal meditation to feel like a ‘*job*’ *(Louise)*. Other obstacles included the repetitiveness of using the same practices, physical discomfort or pain and difficulties concentrating. Due to these challenges, most participants adapted the formal meditation practices to suit them, e.g. listening to the audio-recordings whilst walking the dog, or doing everyday activities mindfully, e.g. washing up. Louise described adopting a mindful ‘*way of thinking*’ into her life. In doing so, several participants touched on their perception that this was perhaps *‘not strictly how you’re supposed to do it’ (John)*.

#### Theme 3: Using mindfulness to respond differently to OCD

This theme captured participants’ perspectives on the ways in which mindfulness helped them to respond differently to OCD.

*Calm and relaxation*. Mindfulness practice made some participants feel calmer or more relaxed. This helped two participants to gain a different perspective on thoughts and to cope with the anxiety of doing ERP.

*Coming back to the present*. Practising mindfulness helped some participants to bring themselves back to the present moment, e.g. coming back to sounds, sights and touch sensations, particularly when they became overwhelmed by intrusive thoughts, associated anxiety and urges to engage in compulsive behaviours. Emma noted this reduced the frequency of her obsessional intrusions. Others similarly described that coming back to the present e.g. by focusing on the breath, interrupted unhelpful repetitive thinking, prevented worrying thoughts from spiralling out of control and helped the mind to ‘*clear*’ *(Hannah)*. Some participants found that coming back to the present reduced their compulsions “*because sometimes I do it [compulsive checking] on autopilot and therefore I would go back to it because I couldn’t actually remember whether I’d done it or not*.*’ (Sarah)*.

*Observing and allowing*. Mindfulness helped participants to observe and allow unwanted thoughts, feelings and sensations. This increased awareness of and exposure to intrusions, which could be challenging at first. Emma, Olivia and George referred to avoiding any attempts to resist, control or suppress their intrusive thoughts, which they related to increased acceptance, e.g. ‘*It has taught me how to deal rather than push away the thoughts’ (Emma)*, de-escalation: *‘I don’t let it spiral out of control […] you just ride with the thought rather than just thinking on and on*.’ *(Olivia)*, and the realisation that intrusions were transient: *‘you don’t have to identify with it […] you can kind of observe it and also realise it will pass*.*’ (George)*. John used mindfulness to stay with physical sensations of anxiety until they decreased naturally: ‘[…] *as soon as I feel that cold rush*, *the tightening of the chest*, *I immediately focus in on that […] it’s a really good way of deflating that kind of anxiety spiral’*. Several participants found this ability also helped them engage with ERP, e.g. ‘*if I didn’t want to do any exposure task […] […] you can like observe a sensation but then [*..*] just do it anyway*.*’ (George)* and ‘*kind of ride the anxiety*’ *(John)*.

*Giving less meaning to intrusions*. A few participants described that mindfulness facilitated new insights into their obsessive thought patterns, realising that ‘*thoughts are just thoughts’ (Olivia)*. For example, Sarah described *‘I don’t sort of believe every anxious thought I have is going to become real […] they are not necessarily going to happen just because I’m thinking about them*.’

### Content analysis

Table 4 summarises the mean (%) perceived changes reported by MB-ERP participants, together with the mean (*SD*) ratings for the extent to which they were surprising, likely to have occurred without the therapy and important. The most frequently reported changes included OCD symptom reduction (reported by 71% of participants), followed by increased ability to manage OCD (64%) and reduced anxiety (50%). Mean ratings for these three changes revealed they were considered very important and unlikely without the therapy course.

**Table 4:** Summary of types of changes reported by participants in study 1 (MB-ERP) (N = 14) and the mean (SD) ratings for the expectedness, likelihood and importance of each type of change.

Type of change	*n* (*%*)	Surprise	Likelihood	Importance
		*M (SD)*	*M (SD)*	*M (SD)*
OCD symptom reduction	10 (71)	3.35 (1.29)	1.56 (0.53)	4.22 (0.71)
Perceived ability to manage OCD	9 (64)	3.50 (1.12)	1.5 (0.50)	3.94 (1.02)
Reduced anxiety	7 (50)	3.57 (1.40)	1.86 (0.38)	4.21 (0.39)
Increased (confidence in) mindfulness skills (to deal with OCD)	5 (36)	3.20 (1.64)	1.60 (0.55)	4.25 (0.96)
Greater awareness or understanding of OCD	4 (29)	4.25 (0.50)	1.63 (1.25)	4.75 (0.50)
Feeling less isolated	4 (29)	3.75 (1.26)	1.50 (0.58)	4.25 (0.50)
Improved mood	4 (29)	3.25 (1.50)	2.25 (1.50)	4.33 (1.15)
Ability to tolerate or manage unpleasant feelings	2 (14)	3.00 (1.41)	1.50 (0.71)	4.00
Greater self-compassion or less perfectionism	1 (7)	4.00	1.00	5.00
Deterioration in mood	1 (7)	4.00	3.00	3.00
Functional improvement	1 (7)	4.00	2.00	4.00
Improved communication	1 (7)	5.00	1.00	5.00

*Note*: *n* = number of participants reporting this type of change, *%* = number of participants reporting this type of change/total number participants.

Surprise = extent to which this change was surprising (1 = ‘very much expected the change to happen’ to 5 = ‘very much surprised by the change’), Likelihood = likelihood that this change would have occurred without the therapy (1 = ‘very unlikely without the therapy course’ to 5 = ‘very likely without the therapy course’); Importance = perceived importance of the change (from 1 = ‘not at all important’ to 5 = ‘extremely important’).

## Methods and materials study 2

### Design and procedure

Interview data was collected as part of an uncontrolled feasibility study of MBCT adapted for OCD. The course was offered to adults with OCD who did not wish to engage in (further) routine CBT (i.e. ERP combined with cognitive strategies) and/or still experienced clinically significant symptoms of OCD after completing CBT. The feasibility study was not pre-registered.

All participants were invited to take part in the Change Interview two to four weeks post-treatment. All interviews were conducted by a research assistant independent of therapy delivery, on NHS premises or at the participant’s home. Interviews were audio-recorded and were 30–60 minutes long. This research project received full ethical approval by an NHS Research Ethics Committee.

### Participants

Participants were recruited through an NHS IAPT service in South East England. Inclusion criteria were as follows: i) aged 18+; ii) met diagnostic criteria for OCD, based on the Mini International Neuropsychiatric Interview, MINI, version 6.0.0 [61] and scored above the clinical cut-off (40 +) on the distress sub-scale of the Obsessive-Compulsive Inventory (OCI) [70]; iii) scored above the clinical cut-off (>16) on the Yale-Brown Obsessive-Compulsive Scale (Y-BOCS) [71] at the baseline research assessment meeting; iv) willing to refrain from another form of psychological therapy during the study; and v) sufficient English language ability to take part in the intervention and complete the study measures. Exclusion criteria were: i) organic cause for OCD; ii) likely diagnosis of learning disability, autistic spectrum disorder, psychosis, anorexia, bipolar disorder, PTSD or reporting a previous diagnosis of psychosis; iii) hoarding-only compulsions; iv) severe symptoms of depression, i.e. score 20+ on the Patient Health Questionnaire-9 (PHQ-9) [72] at the IAPT assessment and/or latest administration of the PHQ-9; v) recorded as presenting a medium to high risk to self or others on the IAPT risk assessment tool; and vi) concerns raised by the assessing clinician about the patient’s suitability for a group intervention.

All seven participants met a diagnosis of OCD at the start of treatment. Mean depression severity of the sample was in the moderate range (*M* = 12.43, *SD* = 2.38), as measured with the PHQ-9 [72]. Two participants were recruited after completing a course of CBT within IAPT and five participants were recruited at the point of IAPT assessment. One of the latter participants had no prior experience of CBT. Among the six participants with prior CBT experience, four completed at least one course of routine CBT treatment whilst one participant had started but prematurely discontinued CBT treatment. See Table 5 for further sample characteristics.

**Table 5:** Sample characteristics for study 2 (MBCT for OCD) (N = 7).

Variable		*M (SD)*	*n*(%)
Age (years)*		39.29 (18.72)	
Age of onset (OCD)*		10.71 (4.54)	
Female			2 (29)
White British			5 (71)
Education	Up to secondary (≤ 12 years education)		6 (86)
	Higher education (university, 12+ years)		1 (14)
Employment	(Self-)employed		5 (71)
	Unemployed		2 (29)
On psychotropic mediation at baseline			4 (57)
(self-reported) Prior CBT	Started		6 (86)
	Completed (at least 1 course of NHS routine treatment for OCD)		5 (71)
No of MBCT-OCD sessions attended		7.00 (1.50) range:4–9	
Dropout			1 (14) ^a^

Note:

^a^ after 4 sessions (including introductory session).

The mean pre-to post-change in Y-BOCS scores was .43 (*SD* = 3.29, 95% CI [-2.01, 2.87], range = -3 - +6) with 1 patient (who completed the intervention) in remission (Y-BOCS ≤ 12 [73] (also see discussion). Treatment completion was defined as attending at least 4 of the main 8 sessions, consistent with MBCT guidelines [31].

### Interview schedule

The Change Interview [63] was also used in study 2. The research team added a further section (9, see S1 Appendix) to the interview schedule, after a lived experience advisory panel recommended them to clarify participant views on whether the intervention facilitated a different way of relating to OCD symptoms, even in the absence of significant symptom reduction.

### Intervention: MBCT-OCD

The MBCT for OCD course (MBCT-OCD) was adapted from the standard MBCT course for recurrent depression [31], in consultation with a lived experience advisory panel. Adaptations included adding an introductory session to the standard 8 weekly 2-hour group sessions and changing the psychoeducational content and cognitive exercises to suit OCD. Psychoeducation included discussing how mindfulness could be used to break the vicious cycle of OCD and was reiterated in each session. In the final two sessions, participants were encouraged to develop an individual action plan and signposted to NHS (e.g. drop-in mindfulness sessions)and other mindfulness resources (e.g. bibliotherapy). See S2 Appendix for course structure and sessional content.

Participants were encouraged to practise formal mindfulness meditations daily (6 days out of 7), aided by standard audio-recordings, and log them on home practice monitoring sheets [31]. Following consultation with an experienced MBCT supervisor, participants who experienced persistent difficulties in finding time to practise were given the option to use short versions (10–15 minutes) drawn from standard MBCT self-help [74] (these resources were made available to all participants from the third session onwards). Participants were also encouraged to apply mindfulness to everyday activities. Between-session tasks included occasional written exercises, as per standard MBCT. All participants were given a workbook containing (adapted) handouts from the standard MBCT course and standard CBT manual used in the IAPT service. The course was facilitated by two experienced clinical psychologists (TL and CS), one of whom was an accredited Cognitive Behavioural Therapy therapist and MBCT teacher (CS). The course facilitators accessed supervision from an accredited MBCT supervisor.

### Data analysis

The thematic analysis and content analysis were conducted in line with the procedure outlined for Study 1.

## Results study 2

### Thematic analysis

Table 3 shows the main and sub-themes that were developed through the thematic analysis. Sub-themes are discussed below. Pseudonyms were used to protect participant confidentiality.

#### Theme 1: Satisfaction with course features

The MBCT-OCD course was generally well-received. Participants described it as, e.g. helping ‘*very quickly’(Mia)* and ‘*super useful’ (Peter)* and reported a range of positive changes and benefits (see content analysis). However, some limitations were also noted and not all participants found the course beneficial.

*Mindfulness approach*. Participants commented on the invitational language used by the facilitators (and in the workbook and audio-recordings). Mia found this really helped her to reduce self-criticism when she ‘*felt like a naughty child’* for not practising mindfulness at home. This contrasted with Mark’s perception that it conveyed *‘a lack of urgency’*, explaining further: *‘I need a certain firmness […] in the initial stages*.*’*. George struggled to grasp the non-striving stance of mindfulness, e.g. when practising the body scan: *‘[…] ‘if you’re meant to get some particular thing out of something*, *there has got to be a way of doing it*, *you’re supposed to feel something*.’.

*MBCT-OCD compared to CBT*. Six participants had (some) previous experience of CBT (ERP with added cognitive strategies). Whilst one participant liked the course precisely because it was an alternative to ERP, most participants described the combination of previous CBT and MBCT-OCD as useful, beneficial or *‘perfect’ (Peter)*. Several participants doubted that mindfulness alone would sufficiently target and reduce OCD symptoms and perceived that it complemented CBT by reducing anxiety associated with intrusions or by bringing an observing stance to intrusions and associated distress: *‘it’s almost like a left brain and a right brain approach’ (Peter)*. They felt that CBT helped to challenge their thinking, which mindfulness might not achieve in isolation: ‘*because I wouldn’t know how to control my thoughts*, *what the logical thinking was*.’ *(Thomas)*. He also thought that mindfulness was not sufficiently direct in targeting OCD symptoms when triggered: *‘in that moment I don’t think it would do much’*, explaining further: *‘you kind of need to know how to rationalise it in the first place’*. George had not found CBT useful in the past, but his difficulties with engaging in mindfulness helped ‘*the penny to drop’* that he could only reduce his OCD symptoms by ‘*facing the fear’*, through self-directed ERP.

*Course and session structure*. Some participants commented that the course was well-structured, with a good balance between well-explained, systematic mindfulness practices and opportunities for discussion and reflection. However, a few participants felt that the course did not allow enough time to share and discuss individual struggles with OCD and others would have liked further sessions to consolidate their mindfulness meditation routine to increase the benefits.

*Workbook and forms*. Some participants found the workbook a well-structured, useful resource, e.g. to catch-up after missing a session, re-visit course content, recall the weekly home practice, and to motivate themselves and consolidate learning: *‘They […] gave a kind of impetus to carry out the work out of sessions […] I like to read something as well as listen to something being explained*.*’(Mark)*. However, a couple of participants found the workbook too comprehensive and complicated, which left them feeling ‘*overwhelmed’ (Mia)*. George felt guilty for *‘[…] not studying the book page to page’*. These participants also found filling in home practice forms or (occasional) written exercises very challenging. They related these difficulties to OCD symptoms that centred on perfectionism and/or feelings of guilt or the impact OCD had on their concentration. Suggestions included simplifying and shortening the manual and including further visual information.

*Therapist embodiment of mindfulness*. All participants commented on the course facilitators, who were perceived as ‘*kind*’ *(Mia)*, ‘*friendly*’ *(Peter)*, ‘*approachable*’*(Mark)* and ‘*trying to understand*’ *(Helen)*, including in relation to participants’ struggles with home practice and/or attendance, e.g. *‘I don’t normally experience that*, *like if I haven’t done something or supposed to be somewhere*, *normally I put on a bulletproof vest and go deal with it [laughs]*.*’ (Peter)*. The therapists’ perceived compassionate stance supported participants to become more self-compassionate, e.g. ‘*certain things that they would say would make me think and realise and now I hear those words’ (Mia)*. The fact that therapists took part in the meditation practices themselves was also positively received: *‘It gave the group a more integrated feel […] it also made me feel as though they were all more invested in the experience and also that […] they believed in the practices themselves’ (Mark)*.

#### Theme 2: Acceptability of key therapeutic tasks

*(Noticing) reactions to formal mindfulness practice*. All participants noticed reactions during formal mindfulness practices, including boredom, frustration, annoyance, concentration difficulties and mind-wandering, confusion, anxiety, (physical) discomfort, sleepiness and exhaustion, feeling lost or overwhelmed, relaxation and enjoyment. These reactions occurred both within sessions and at home and were sometimes noted in relation to specific practices (see below). Struggles to concentrate could be stressful or frustrating and at times resulted in impulses, not necessarily acted upon, to stop the meditation and engage in other activities, particularly when at home. Feelings of irritation and frustration emerged in response to the (standard MBCT) audio recordings; several participants disliked or lacked ‘*affinity*’ *(Mark)* with the voice, finding it ‘*robotic*’ *(Thomas)*, ‘*annoying*’ *(Helen)* or ‘*off-putting’ (Thomas)*, or felt there weren’t enough pauses in the guidance. Participants were encouraged to kindly observe any unpleasant thoughts, feelings or sensations or discomfort they noticed, which Helen reflected on in relation to mind wandering: ‘*knowing that your mind wanders*, *but just bring it back*, *the more you practise that kind of thing*, *the easier things will get*.*’*.

*Preference for type of formal practice*. Several participants talked about disliking and/or struggling with the body scan meditation, finding it too long and challenging. Paying attention to parts of the body during the body scan could bring heightened awareness of painful sensations and physical discomfort, which for one participant triggered OCD symptoms. Conversely, experiencing little or no sensation during the body scan generated frustration, boredom and sleepiness, mind wandering and difficulties concentrating or focusing. Some participants experienced the body scan as valuable, relaxing and calming and persisted with this practice at home. Others expressed preferences for sitting practices with a single focus, e.g. on the breath, or movement practices, which they found easier to concentrate on. The length of formal practice was often discussed; most participants found it easier to focus on shorter practices, fit them into daily life, e.g. whilst on the bus or at work, or build them into a routine. Participants also found it easier to engage with bringing mindful awareness to everyday activities.

*(Struggles with) developing a mindfulness practice routine*. Most participants realised that developing mindfulness skills takes time and effort and that they were more likely to reap benefits with regular practice. They expressed a wish to get in the ‘*flow*’ *(Mia)* and ‘*schedule*’ mindfulness practice rather than it being ‘*on and off’ (Thomas)* but also voiced various struggles with developing a regular mindfulness routine during and after the course. Participants’ reactions to being set home practice included feeling it was *‘formulaic’ (Thomas)* or that there were too many set practices. Helen also described the human tendency to ‘*object*’ to anything that you are set to do, through connotations with ‘*homework*’ *(Mia)*. Participants often felt they should practise more or longer than they did. Obstacles included ongoing health problems, distractions, other commitments, difficulties prioritising practice and finding an appropriate place to practise. Considering these obstacles, several participants chose to engage in the shorter (versions of) formal practices or prioritised bringing mindfulness to everyday life, e.g. walking the dog, even if some perceived that this was not quite the ‘right’ way to practise.

*Comparing mindfulness practice at home and in session*. Most participants found it easier to practise mindfulness in session, without the usual distractions and other commitments at home. Having made time for the session or feeling that it would not possible to ‘*make excuses*’ *(Mia)* helped participants to immerse themselves in the practices. Several participants had a more favourable reaction to ‘live’ guidance than audio-recordings, finding it much more engaging. However, one participant preferred practising alone, in the comfort of his own home.

#### Theme 3: Using mindfulness to respond differently to OCD

*Becoming (more) aware*. Mindfulness helped some participants to become more aware of intrusions and self-critical thoughts and how these tend to escalate, e.g. ‘*I realise quickly when I interact with my OCD’ (Robert)*. This could be difficulty initially, e.g. Mia described it increased her awareness of the extent of her self-critical thoughts. Increased awareness of OCD was related to an ability to (sometimes) disengage from it and ‘*eventually stop the cycle*.*’ (Robert)*. Peter described a general increase in body awareness, including physical sensations associated with anxiety, ‘*I can actually feel the adrenaline more’*, which helped him to regulate his emotions. Bringing mindful awareness to everyday activities such as washing up also helped him to ‘c*ome back to normal very quickly*’ when he felt unsettled.

*Coming back to the present*. A few participants reflected on how mindfulness helped them to come back to the present moment, e.g. coming back into the body when experiencing anxiety. This had a calming effect and allowed a degree of detachment from unhelpful thinking processes, seeing them from a different perspective: ‘*what goes on in your head […] doesn’t seem as real anymore’ (Robert)*. Focusing on ‘*the here and now’ (Peter)* also benefitted other mental health difficulties, including depression and coping with painful memories, because *‘[…] you understand everything is like memories or future projections*, *it’s not actually happening*.*’(Peter)*.

*Observing and allowing*. Several participants commented on their ability to observe unpleasant experiences with a degree of detachment. This ‘*observer status of mind’ (Peter)* or ability to ‘ *step back’ (Helen)* and ‘*look at it from a distance’ (Robert)* was considered an important skill that could be applied to intrusions, anxiety and depressed mood. Allowing difficult feelings at times heightened their intensity because, e.g. ‘*I’m not hammering them away or ignoring them or doing something to get rid of them’ (Peter)*. Over time, the process of observing difficult feelings could to reduce their intensity. Being able to observe and allow intrusions also contributed to participants’ ability to respond differently to compulsions, e.g. allowing thoughts to become more ‘*rational’ (Thomas)* and dealing with compulsions more calmly. Peter connected it to an ability to pause and reflect on compulsive urges and make a choice how to respond: *‘I can either do that compulsion knowing full well that it’s a compulsion or I can not do it and get on with my life’*. This was a challenging, time-consuming process that was not always achievable when compulsions were pervasive.

*Giving less meaning to intrusions*. Several participants reflected that noticing unpleasant thoughts, coming back to the present or allowing them to remain in awareness and observing them with kindness, facilitated a change in perspective on intrusions, or a ‘*different point of view*’ *(Helen)*. Robert also described this process of looking at intrusions ‘*from a different perspective’* as *‘detaching from those thoughts’*, realising *‘I don’t have to pay attention to it’* and *‘you don’t have to react to them’*, whilst Helen experienced that ‘*standing back’* allowed her to ‘*not give so much meaning to everything that’s in your head*.*’*

*Calm and relaxation*. A few participants experienced that mindfulness practice had a calming, relaxing effect on the body and allowed them to be *‘more logical’ (Thomas)* and think more clearly. Thomas described this as a first step in re-evaluating his thoughts, which he related to skills learnt during CBT. It also allowed him to have more control over compulsions. Peter also linked mindfulness practice to turning *‘the volume of the anxiety right down’* and to a cessation of ‘*mental chatter’*.

*Kindness to self*. Some participants who struggled with perfectionism and self-critical thoughts really connected with the ‘*kindness*’ or ‘*nonjudgment*’ and ‘*being curious*’ that mindfulness invited them to bring to their intrusions. This helped them not to get caught up in the OCD cycle: *‘[…] I might say ‘Okay*, *this is you being unkind to yourself*, *remember to be kind’ and then it moves on*. *So*, *yes*, *it doesn’t sort of escalate*.*’ (Mia)*. The ability to be kind to oneself was also noted as a consequence of coming back to the present: ‘*cos it’s just kind of seeing what’s real*, *as in like*, *it’s the present and that’s it*, *that’s all that matters’ (Robert)*. Peter also felt that responding to depressed mood with kindness helped to alleviate it.

### Content analysis

Table 6 summarise the results of the content analysis. The main reported changes were reduced anxiety (reported by 71% of participants) followed by OCD symptom reduction and increased ability to manage OCD (both 43%). Ratings for these changes suggested that the changes were considered very important and unlikely without the therapy course.

**Table 6:** Summary of types of changes reported by participants in study 2 (MBCT for OCD) (N = 7) and the mean (SD) ratings for the expectedness, likelihood and importance of each type of change.

Change	*N* (*%*)	Surprise	Likelihood	Importance
		*M (SD)*	*M (SD)*	*M (SD)*
Reduced anxiety	5 (71)	2.40 (1.52)	1.80(0.84)	4.80 (0.45)
OCD symptom reduction	3 (43)	2.67 (1.15)	1.67 (0.58)	4.67 (0.58)
Perceived ability to manage OCD	3 (43)	3.50 (2.12)	1.67 (1.15)	4.67 (0.58)
Greater awareness or understanding of OCD	2 (29)	3.50 (0.71)	2.50 (1.41)	5.00
Greater self-compassion or less perfectionism	2 (29)	3.50 (0.71)	1.50 (0.71)	4.50 (0.71)
Feeling less isolated	2 (29)	2.00	2.00	3.50 (0.71)
Ability to manage other mental health problems	1 (14)	5.00	1.00	5.00
Increased (confidence in) mindfulness skills (to deal with OCD)	1 (14)	3.00	2.00	4.00
Deterioration in mood	1 (14)	5.00	3.00	1.00
Improved sleep	1 (14)	3.00	3.00	3.00
Motivation to overcome OCD	1 (14)	4.00	1.00	5.00
Random anxiety	1 (14)	3.00	3.00	1.00

*Note*: *N* = number of participants reporting this type of change, *%* = number of participants reporting this type of change/total number participants. Surprise = extent to which change was surprising (1 = ‘very much expected the change to happen’ to 5 = ‘very much surprised by the change’), Likelihood = likelihood that change would have occurred without the therapy (1 = ‘very unlikely without the therapy course’ to 5 = ‘very likely without the therapy course’); Importance = perceived importance of the change (from 1 = ‘not at all important’ to 5 = ‘extremely important’).

## Discussion

### Summary of results

This paper reports on two consecutive studies that explored participants’ perceptions of the acceptability, potential mechanisms and benefits of MBIs that integrated mindfulness with CBT in different ways. The reflexive thematic analyses of the Change Interview data for study 1 (MB-ERP) and 2 (MBCT-OCD) had all three main themes in common, i.e. ‘satisfaction with course features’, ‘acceptability of key therapeutic tasks’ and ‘using mindfulness to respond differently to OCD’. Most participants in both MB-ERP and MBCT-OCD were satisfied with their course and considered it an acceptable treatment for OCD. This discussion will first discuss unique sub-themes for study 1 and 2, respectively, followed by common sub-themes across both studies, before discussing the content analysis of participant reported changes, limitations of the study and recommendations for future research.

#### Sub-themes unique to study 1 (MB-ERP)

Most participants liked the integration of mindfulness with ERP and felt it had added benefit to ERP and psychoeducation. This resonates with the theoretical premise for using mindfulness to enhance exposure, e.g. [46], and the views of the lived experience advisory panel consulting to the study.

#### Sub-themes unique to study 2 (MBCT-OCD)

Most participants had prior experience of CBT for OCD and conveyed a positive orientation towards CBT. They appeared to find the combination of MBCT with CBT beneficial, suggesting these approaches complemented each other well and that the two in combination might lead to improved outcomes than either on its own. The invitational, non-striving stance of mindfulness received a mixed reception and participants questioned the potency of mindfulness as a stand-alone therapy for OCD. Among the couple of participants who were less favourably disposed towards ERP, there was a mixed response to MBCT-OCD; it helped one participant to develop the capacity for self-compassion to benefit OCD symptoms centred on perfectionism, whereas the other participant did not feel able to engage with MBCT-OCD and concluded that ERP would now be a preferable treatment.

Whilst the course structure and content was generally well-received, some participants wanted further sessions to consolidate their skills. Also, MBCT is an experiential, process-orientated group intervention [31]; some participants wanted more opportunities to discuss individual OCD symptoms. Participant reflections on the therapists’ kindness and understanding highlights the importance placed on the ‘embodiment’ of mindfulness by course facilitators of MBCT [31]. Seemingly more peripheral features of the course, i.e. workbooks and forms, also had the potential to influence engagement.

Whilst many participants wished to establish a sustained mindfulness practice, many noticed aversion or attachment to certain experiences during mindfulness meditation. These experiences are ‘par for the course’ and provide fertile soil for learning to develop an accepting, kinder attitude towards such experiences rather than interpreting this as a personal failure or incompetence [31]. However, this is undoubtedly challenging and some participants opted for shorter or informal mindfulness practices rather than continuing with practices that gave rise to unpleasant experiences.

The issues raised by participants in relation to the potency and duration of the course and the opportunity for discussion of individual OCD symptoms were also reported by Sguazzin et al. [58], as were impatience, being put off by the voice in audio recordings and lacking motivation as obstacles to mindfulness practice. These issues may not be unique to OCD, e.g. Mason and Hargreaves [75] touch on similar ‘initial negative experiences’ in their study of MBCT for depression while participants with current depression or anxiety thought the MBCT course was too short and similarly struggled with longer practices, particularly the body scan [76]. A meta-synthesis of themes from 15 qualitative studies of group MBIs for a range of mental health problems included sub-themes, i.e. ‘biggest challenge’, ‘mix and match’ and ‘I focused on having to achieve something’, that highlighted similar issues [77].

Overall, participant responses to the course content and structure also brought home the complexities of understanding and untangling the role of both treatment-specific and common factors, patient sociodemographic, clinical and/or psychological characteristics in understanding patient engagement [78–80].

#### Common sub-themes across study 1 and 2

Mindfulness-based interventions attach key importance to home practice [81], to develop and consolidate mindfulness skills [31]. MBCT-OCD participants were invited to engage in longer practices (both within- and between sessions) compared to MB-ERP participants. The sub-theme ‘(struggles with) developing a mindfulness practice routine’ nonetheless highlighted challenges that both MB-ERP and MBCT-OCD participants faced in engaging (consistently) with the recommended formal mindfulness practice at home. This does not appear to be unique to people with OCD as similar challenges were noted by participants in MBCT for current depression or anxiety [76] and recent meta-analyses found that participants with a range of physical and mental health problems did not necessarily fully adhere to the recommended weekly mindfulness practices [82, 83]. Many participants established personalised ways of practising mindfulness that were perceived as more accessible and suited their lifestyles. This resonates with the finding in Hertenstein et al. [57] that participants modified practices to suit their needs. Some participants felt that their OCD symptoms prevented them from engaging in mindfulness practice. Sguazzin et al. [58] and Hertenstein et al. [57] also reported that OCD symptoms were perceived to ‘conflict’ with practice and/or were all-consuming, not dissimilar to research showing that depressive rumination can affect engagement in MBCT for (recurrent) depression [84]. As consistent formal home mindfulness practice is associated with the alleviation of depression and anxiety symptoms, e.g. [82, 83], the current findings, i.e. that participants did not necessarily feel able to engage consistently with recommended formal mindfulness practice, complicates the consideration of the potential efficacy of MBIs for OCD.

Both MB-ERP and MBCT participants reflected on similar, inter-related, ways in which mindfulness helped them to relate differently to obsessional intrusions and compulsions. This included: i) ‘coming back to the present’; ii) ‘observing and allowing’; iii) ‘giving less meaning to intrusions’; and iv)‘calm and relaxation’. MBCT-OCD participants also noted; v) (becoming more aware) of OCD (closely associated with subtheme ii)) and; vi) kindness to self, particularly in relation to OCD symptoms that centre on (negative) perfectionism (e.g. [9]). MB-ERP participants also appeared to support the proposition that mindfulness could aid engagement with ERP (Treanor, 2011) and this resonates with another qualitative evaluation of a similar integration of mindfulness with CBT [59].

In ERP the evocation of anxiety (through presenting an appropriate trigger for obsessional intrusions) is necessary to facilitate emotional processing [4] and/or inhibitory learning [6]. Therefore, the perceived calming, relaxing effect of mindfulness practice could potentially function as a distraction or neutralising technique that prevents inhibitory learning during ERP [46] and thereby could be a barrier to overcoming OCD. It highlights that the intention of mindfulness practice in this context should be to facilitate an aware, welcoming and accepting attitude towards intrusive thoughts and associated feelings and body sensations rather than to support disengagement or distraction from intrusive thoughts.

In the current studies, however, participants described that reducing anxiety associated with intrusions through mindful, non-judging, acceptance of these experiences, benefitted wiser choices about how to respond to OCD. The fact that ‘observing and allowing’ appeared to aid exposure to intrusions, associated distress and compulsive urges in ways that benefitted reappraisal of intrusions (reflected in the ‘giving less meaning to thoughts’ sub-theme) also supports the assertion that mindfulness could encourage an accepting awareness of intrusions and associated distress compatible with inhibitory learning theory approaches to ERP (e.g. [28, 59, 85]). The fact that only MBCT-OCD participants noted ‘kindness to self’ may result from the primary emphasis placed on mindfulness practice and inquiry in MBCT-OCD relative to MB-ERP. This perhaps more clearly conveyed the mindfulness approach, which has (self-)compassion at its heart [31]. The fact that MBCT-OCD participants reflected on ‘therapist embodiment of mindfulness’ and the ‘mindfulness approach’, e.g. invitational language and non-striving, also supports this notion.

Together, the ways in which participants reported ‘using mindfulness to respond differently to OCD’ mapped onto theorised mechanisms of action of mindfulness for OCD [43]. The overlap in sub-themes between MBCT-OCD and MB-ERP participants lends further credibility to these findings as does the fact that findings mirrored other qualitative evaluations of mindfulness integrated with CBT (including ERP) (e.g. [59]) and MBCT adapted for OCD [57–59].

### Content analysis

The content analysis highlighted significant overlap in perceived benefits and changes reported by MB-ERP and MBCT participants, despite the fact that MB-ERP foregrounded ERP whilst MBCT emphasised mindfulness as the primary vehicle for change. Changes included a reduction in OCD symptoms (71% of MB-ERP and 43% of MBCT-OCD participants), whilst increased awareness of, and ability to manage, OCD together were also reported by the majority of participants (MB-ERP: 93%, MBCT: 72%). Reduced anxiety was also frequently reported (MB-ERP:50%, MBCT-OCD: 71%). Perceived changes extended beyond OCD to include beneficial effects on other mental health problems, stress reduction and functional improvement. Participants in Hertenstein et al. [57] and Sguazzin et al. [58] similarly reported improved knowledge and understanding of OCD along with functional improvement, reductions in comorbid symptoms, stress relief and general well-being. This lends further credibility to the content analysis.

### Limitations

All participants were recruited through (one of) two psychological therapy services in South East England. This, and the lack of ethnic diversity among participants in MB-ERP, limits the transferability of the findings. Furthermore, there were differences between the study samples that may limit conclusions drawn from comparing the findings of the two studies. MB-ERP participants were recruited at the point of routine IAPT initial assessment, whereas MBCT-OCD participant were recruited during IAPT initial assessment or after completing CBT treatment in IAPT. MB-ERP incorporated ERP, i.e. the NHS recommended treatment for OCD, and, therefore, was offered to all eligible patients irrespective of previous experience of ERP. MBCT-OCD did not include ERP and, therefore, all patients eligible at the point of assessment were made aware that ERP was the recommended treatment and that it was available to them within IAPT. MBCT-OCD was explored as an alternative for those patients with clinically significant OCD symptoms who did not want to engage in (further) routine CBT and who would ordinarily be discharged from IAPT. Consequently, half of the MB-ERP participants had no previous experience of CBT versus only 14% of MBCT-OCD participants. Furthermore, MB-ERP participants were interviewed at follow-up whereas MBCT-OCD participants were interviewed post-treatment. Nonetheless, the convergence of results relating to benefits and potential mechanisms of MB-ERP and MBCT-OCD participants and other qualitative studies on MBIs for OCD lends some support to the potential transferability of the findings.

Evidently, participants in MB-ERP were invited to participate in shorter and less varied formal mindfulness meditation practices (within and between-sessions) compared to participants in MBCT-OCD. Within the MBCT-OCD group of participants, some participants opted for shorter versions of body scan and sitting meditation practices for their home practice. This will have influenced participant perceptions of the potential benefits of these interventions. Nonetheless, MB-ERP and MBCT-OCD participant reports on how mindfulness benefitted their OCD symptoms had much in common, reflected in common themes.

The Change Interview did not explicitly enquire about participants’ perceptions of mindfulness. Specific questions about the (dis)advantages of mindfulness may have generated further information, particularly for MB-ERP participants for whom mindfulness was the smaller component of the course.

The first author was a co-facilitator of the MBCT-OCD course. This has the potential to influence what is inferred from participant reports. Care was taken to stay close to participants’ verbal accounts, represent ‘negative’ aspects highlighted by participants and to reach group consensus on main and sub-themes, clearly derived from initial coding [69].

### Clinical and research implications

The preliminary evidence towards theorised mechanisms of change could be taken forward through experimental research and mediational analyses in the context of treatment studies. Specifically, facets of mindfulness (observing, acting with awareness, nonjudging, nonreactivity) could be explored as potential mechanisms of change. The ‘giving less meaning to thoughts’ and ‘observing and allowing’ subthemes also suggest that concepts that are similar to the nonjudging facet of mindfulness, i.e. reperceiving [86], decentering [87], meta-cognitive awareness [88] and/or thought-action fusion (the belief that having a bad thought is morally equivalent to, or increases the likelihood of, acting on the thought) [89, 90] should be explored as potential mechanisms of change. The ‘kindness to self’ sub-theme for MBCT-OCD suggests that self-compassion skills may help to target OCD symptoms, particularly those that centre on order, symmetry and perfection and/or self-critical thoughts associated with unacceptable (sexual or aggressive) intrusions and feelings of shame [91, 92].

Participants in both MBIs found the combination of mindfulness with CBT beneficial. We speculate that barriers to successful CBT for OCD for some people (e.g. appraising intrusive thoughts as facts, distress intolerance, carrying out compulsions in automatic pilot and lack of self-compassion) could be directly addressed through the integration of a mindfulness-based approach that is intended to target these barriers. Through successfully targeting these barriers, such people may be able to successfully engage in CBT for OCD tasks (e.g. appraising intrusive thoughts as unimportant mental events, distress tolerance, conscious awareness of compulsive urges and self-compassion) and as such have a positive treatment outcome, given that task engagement is key to positive outcomes (Simpson et al, 2011). Therefore, research could further explore the effect of different types of integrations of CBT with mindfulness on OCD symptoms.

The discrepancy between patient satisfaction and personally valued changes on the one hand and a lack of substantive OCD symptom reduction on the other (see participant information above; also see a recent RCT of MBCT as an augmentation therapy for CBT [28]) requires further investigation before drawing more definitive conclusions about the efficacy of delivering MBIs for OCD.

Obsessive-compulsive disorder is a heterogeneous disorder as obsessions can centre on a fear of contamination, (unintentionally) causing or preventing harm to self or others, symmetry or ‘taboo’ thoughts, such as unwanted aggressive, sexual or blasphemous thoughts, images or impulses [93, 94]. OCD symptom subtypes characterised by taboo thoughts and strongly associated with thought-action-fusion beliefs [95] and/or feelings of shame (e.g. [48, 96]) may be less responsive to ERP [97, 98]. Also, OCD symptoms characterised by mental rather than overt compulsions may be more difficult to treat with (imaginal) ERP than overt compulsions [36, 99, 100]. These OCD subtypes may therefore be particularly suitable for the exploration of innovative (add-on) interventions including MBIs, which invite participants to develop a different relationship to their symptoms through mindfulness meditation practice rather than ERP. OCD symptoms characterized by high levels of perfectionism may also derive benefit from MBIs, which place self-compassion is at the heart of the intervention [37, 101, 102]. However, the notion that MBIs may bring differential benefit according to OCD subtype is conjecture at present and requires further exploration.

Whilst research into well-established MBIs such as MBCT and MBSR has not yet included dismantling the effects of mindfulness from common factors/group processes, it will be important to examine the extent to which (lack of) benefits of MBIs for OCD may be attributable to the common factors, such as the group format, or mindfulness meditation practice; this understanding will help to maximise therapy outcomes [52]. Delivering MBIs for OCD in group format may not necessarily be most effective given the heterogeneity of OCD; the idiosyncratic nature of OCD may mean that participants do not necessarily feel that able to generalise from presentations other than their own.

The MBIs studied here might have lacked a more potent effect because most participants reported that they did not necessarily engage in regular, sustained formal mindfulness practice as recommended in the protocols. This could reflect the ‘counter-cultural’ quality of mindfulness approaches [30], transdiagnostic experiential avoidance (e.g. [103]) and/or challenges specific to OCD, e.g. high levels of distress intolerance (e.g. [15]), (2011) or the relentless, consuming nature of obsessional intrusions. Further research is needed into the role of specific and non-specific treatment factors, sociodemographic and/or clinical patient characteristics and psychological processes in treatment response.

Further research could investigate ways of making formal mindfulness practices more accessible. For example, although there is a well-considered rationale for using the body scan as the first meditation practice [31], most participants found this a very challenging meditation to begin with [76]. A more gradual build-up in length of mindfulness practices might help to build distress tolerance [15] and enhance engagement. Increased use of mindful movement practices in the early stages of the course may also benefit engagement as they enable participants to notice physical sensations more readily. Didonna [44] similarly proposed that for people with severe OCD symptoms, it may be helpful to gradually introduce mindfulness. For example, it might be advisable to progress from walking practices (that generate proprioceptive sensations) to the body scan (interoceptive sensations), from short to long exercises and from informal to formal practice. A case study by Patel [104] similarly described introducing mindfulness practices gradually and starting with most preferred/least intrusive practices. Offering practices on preference, e.g. depending on OCD symptom presentation, may help enhance mastery and ease people into committing to daily practice. Evidently, this requires careful consideration as it could potentially lead to avoidance of practices that bring people into more direct contact with difficulty which, whilst unpleasant, may be therapeutically beneficial. However, supporting people to engage with mindfulness practice early on in therapy is arguably worthwhile if this can avoid treatment dropout. Facilitators of MBCT for OCD may also need to (repeatedly) emphasise further that mind wandering and unpleasant sensations or reactions during mindfulness practice are normal and do not signal personal failure, and indeed are helpful as they allow mindful skill development in relation to such experiences (e.g. [31]). As gathering home practice logs proved difficult (a recurrent problem even in well-established MBCT for depression research [101–105]), the use of technology such as apps (which would automatically log patients accessing mindfulness meditation practices) would aid research into engagement in MBIs for OCD [105]. Similarly, it would be beneficial to include post-treatment questionnaires eliciting the extent of home practice. In conclusion, findings suggested that patients with OCD value MBIs in relation to their OCD difficulties but also emphasised the need for further research to understand whether and how their potential to improve OCD symptoms may be enhanced.

## Supporting information

S1 Appendix Change interview adapted from Elliott (2001). (PDF)

S2 Appendix Overview of MBCT session content and structure, as adapted for OCD. (PDF)
