# Supplementary material for: Participant perspectives on the acceptability and effectiveness of mindfulness-based cognitive behaviour therapy approaches for obsessive compulsive disorder
Source: PLoS One. 2020 Oct 21;15(10):e0238845. doi: 10.1371/journal.pone.0238845 (PMC7577499; doi:10.1371/journal.pone.0238845)
Supplement: S1 Appendix — (PDF) [file pone.0238845.s001.pdf]

S1 Appendix

Change Interview

(Adapted from Elliott, 2001)

Interview Strategy: This interview works best as a relatively unstructured empathic exploration of the client's experience of the course. Think of yourself as primarily trying to help the client tell you the story of his or her the course so far. It is best if you adopt an attitude of curiosity about the topics raised in the interview, using the suggested open-ended questions plus empathic understanding responses to help the client elaborate on his/her experiences. Thus, for each question, start out in a relatively unstructured manner and only impose structure as needed. For each question, a number of alternative wordings have been suggested, but keep in mind that these may not be needed.

- Ask client to provide as many details as possible
- Use the "anything else" probe (e.g., "Are there any other changes that you have noticed?"): inquire in a non-demanding way until the client runs out of things to say

Introduction given to clients: After the course, clients are asked to come in for a semi-structured interview that can take up to one hour. The major topics of this interview are any changes you have noticed since the course began, what you believe may have brought about these changes, and helpful and unhelpful aspects of the course. The main purpose of this interview is to allow you to tell us about the course and the research in your own words. This information will help us to understand better how the course works; it will also help us to improve the course. This interview is audio-recorded for later transcription. Please provide as much detail as possible.

Interview Schedule:

1. ***Changes***: [about 10 min]
1a. What changes, if any, have you noticed in yourself since the course started? *(Interviewer: Reflect back change to client and write down brief versions of the changes for later. If it is helpful, you can use some of these follow-up questions: For example, Are you doing, feeling, or thinking differently from the way you did before? What specific ideas, if any, have you gotten from the course so far, including ideas about yourself or other people? Have any changes been brought to your attention by other people?)*

1b. Has anything changed for the worse for you since the course started?

i. ……………………………………………………………………………………………

ii. ……………………………………………………………………………………………

iii. ……………………………………………………………………………………………

iv. ……………………………………………………………………………………………

v. ……………………………………………………………………………………………

1c. Is there anything that you wanted to change that hasn't since the course started?

i. ……………………………………………………………………………………………

ii. ……………………………………………………………………………………………

iii. ……………………………………………………………………………………………

iv. ……………………………………………………………………………………………

v. ……………………………………………………………………………………………

2. ***Change Ratings***: [about 10 min] (Go through each change and rate it on the following three scales:)
2a. For each change, please rate how much you expected it vs. were surprised by it? (Use this rating scale:)

(1) Very much expected it
(2) Somewhat expected it
(3) Neither expected nor surprised by the change
(4) Somewhat surprised by it
(5) Very much surprised by it

2b. For each change, please rate how likely you think it would have been if you hadn't done the course? (Use this rating scale:)

(1) Very unlikely without the course (clearly would not have happened)
(2) Somewhat unlikely without the course (probably would not have happened)
(3) Neither likely nor unlikely (no way of telling)
(4) Somewhat likely without the course (probably would have happened)
(5) Very likely without the course (clearly would have happened anyway)

2c. How important or significant to you personally do you consider this change to be? (Use this rating scale:)

(1) Not at all important
(2) Slightly important
(3) Moderately important
(4) Very important
(5) Extremely important

	Expected it? (1-5)	Likely? (1-5)	Importance? (1-5)
Change 1:			
Change 2:			
Change 3:			
Change 4:			
Change 5:			

3. ***Attributions***: [about 5 min] In general, what do you think has caused the various changes you described? In other words, what do you think might have brought them about? (Including things both outside of the course and in the course)

4. ***Helpful Aspects***: [about 10 min] Can you sum up what has been helpful about the course so far? Please give examples. (For example, general aspects, specific events)

5. ***Resources***: [about 5 min]
5a. What personal strengths do you think have helped you make use of the course to deal with your problems? (what you're good at, personal qualities)

5b. What things in your current life situation have helped you make use of the course to deal with your problems? (family, job, relationships, living arrangements)

6. ***Problematic Aspects***: [about 5 min]
6a. What kinds of things about the course have been hindering, unhelpful, negative or disappointing for you? (For example, general aspects. specific events)

6b. Were there things in the course which were difficult or painful but still OK or perhaps helpful? What were they?

6c. Has anything been missing from your treatment? (What would make/have made the course more effective or helpful?)

7. ***Limitations***: [about 5 min]
7a. What personal limitations do you think have made it harder for you to use the course to deal with your problems? (things about you as a person)

7b. What things in your life situation have made it harder for you to use the course to deal with your problems? (family, job, relationships, living arrangements)

8. ***Suggestions***. [about 5 min] Do you have any suggestions for us, regarding the research or the course? Do you have anything else that you want to tell me?

9. ***Final reflections*** [about 5-10 minutes] on the course (**MBCT course only**):

9a) What were the main issues or difficulties you were experiencing *before* you started the course?

9b) To what extent have these issues or difficulties changed since undertaking and completing the course?

9c) How do you live with these issues or difficulties now? Have you noticed any differences in how you live with these issues or difficulties since completing the course?

Rating Scales:

**1**	**2**	**3**	**4**	**5**
Very much expected the change to happen	Somewhat expected the change to happen	Neither expected the change to happen nor was surprised by it	Somewhat surprised by the change	Very much surprised by the change

**1**	**2**	**3**	**4**	**5**
Very unlikely without the course (clearly would not have happened)	Somewhat unlikely without the course (probably would not have happened)	Neither likely nor unlikely (no way of telling)	Somewhat likely without the course (probably would have happened)	Very likely without the course (clearly would have happened anyway)

**1**	**2**	**3**	**4**	**5**
Not at all important	Slightly important	Moderately important	Very important	Extremely important
