# Supplementary material for: Participant perspectives on the acceptability and effectiveness of mindfulness-based cognitive behaviour therapy approaches for obsessive compulsive disorder
Source: PLoS One. 2020 Oct 21;15(10):e0238845. doi: 10.1371/journal.pone.0238845 (PMC7577499; doi:10.1371/journal.pone.0238845)
Supplement: S2 Appendix — (PDF) [file pone.0238845.s002.pdf]

# S2 appendix

*Overview of MBCT session content and structure, as adapted for OCD*

| **Session no. & theme** | **Content** | **Practices** |
|---|---|---|
| - Introductory | Introductions; ground rules; introducing the cognitive model of OCD; how MBCT might help people with OCD; what taking part in the course involves | Introductory mindfulness practice (breathing/grounding) |
| 1. Awareness and automatic pilot | Re-visiting introductions and ground rules; selective attention/hypervigilance in OCD; getting caught up in (worrying about & responding to) intrusive thoughts | Marble exercise; body scan; everyday mindfulness, 2-3 minute breathing practice |
| 2. Living in our heads | Normalising intrusive thoughts; setting up pleasant events calendar practice | body scan, 10-minute sitting meditation |
| 3. Gathering the scattered mind | Inviting participants to use the 3-minute breathing space (3MBS) when OCD symptoms emerge, setting up unpleasant (mental) events calendar | 5 minute ‘seeing’/ ‘hearing’ exercise; 30-minute sitting meditation; 3-minute breathing space (3MBS); Mindful stretching |
| 4. Recognising aversion | Defining the territory of OCD (revisiting the cognitive model); the role of (aversion to) anxiety; | 5-minute ‘seeing’/‘hearing’ exercise; 30- to 40- minute sitting meditation; poem, e.g. ‘wild geese’; 3MBS; Mindful walking |
| 5. Allowing, letting be | Exploring the meaning we give to intrusive thoughts (i.e. obsessive beliefs) using the **OBQ-20**<br>The role of thought suppression, avoidance and compulsions in maintaining OCD | 30- to 4- minute sitting meditation; introducing a difficulty within the practice and noting its effects on the body and reactions to it; Breathing space (with added instructions); Rumi’s poem ‘the guest house’ |
| 6. Thoughts are not facts | Preparation for end of course; discuss breaking the OCD vicious cycle; Introduce the notion of ‘Theory A vs Theory B’; discuss distress/anxiety tolerance and building up confidence that one can tolerate distress/anxiety | 30-to 40- minute sitting meditation, noticing how we relate to thoughts that arise, Breathing space |

| | | |
|---|---|---|
| 7. How can I best take care of myself? | Using the 3MBS as a first step before choosing how best to respond to OCD; self-compassion vs immediate relief in overcoming OCD; keeping up the good work | 30- to 40- minute sitting meditation – awareness of when difficulties arise within the practice, noting their effects and reactions to them, on the body, 3MBS or mindful walking |
| 8. Maintaining and extending new learning | Review of the course and what has been learnt; personal reflections; how to keep up momentum | Body scan practice, concluding meditation (marble, stone or bead)/participants wishing each other well |

S
